# Comment on “Hepatectomy Versus Sorafenib in Advanced Nonmetastatic Hepatocellular Carcinoma”

**DOI:** 10.1097/AS9.0000000000000254

**Published:** 2023-01-30

**Authors:** Xinye Qian

**Affiliations:** From the Center of Hepatobiliary Pancreatic Disease, Beijing Tsinghua Changgung Hospital, School of Clinical Medicine, Tsinghua University, Beijing, China.

Hepatocellular carcinoma (HCC) with portal vein tumor thrombus (PVTT) is a difficult disease, associated with the development of metastatic nests, and higher incidence in tumors exhibiting microvascular invasion and/or satellite lesions.^1^ Although systemic therapy is still recommended for HCC with PVTT in the 2022 version of the Barcelona Clinic Liver Cancer (BCLC) staging and treatment strategy,^2^ the outcome for these patients are not ideal even with the latest Atezolizumab-Bevacizumab therapy (median Progression Free Survival (PFS)month for patients with HCC and PVTT is 14.2).^3^ Meanwhile, Famularo et al^4^ reported, in their work “Hepatectomy Versus Sorafenib in Advanced Nonmetastatic Hepatocellular Carcinoma,” that liver resection is associated with increased survival in patients with advanced, intrahepatic macrovascular invasive, nonmetastatic HCC when compared with sorafenib treatment (median PFS month for HCC patients with PVTT underwent surgery is 24), which is exciting. Other evidence also supports the idea that surgical resection should be considered in selected patients with resectable HCC and PVTT.^5^ We have witnessed the 2022 version of BCLC staging and treatment strategy updated the subgroup of BCLC B patients and extended liver transplant criteria for HCC patients. With the accumulation of evidence, we could expect that strategy for patients with HCC and PVTT might change in the near future.

In many similar studies, neoadjuvant therapy was applied before surgical resection, which might affect the result of these studies. This is because only HCC patients responded to the neoadjuvant therapy would receive surgery. Simple math would tell us the survival of patients, all of who respond to neoadjuvant therapy, is better than the survival of patients, only a part of who respond to neoadjuvant therapy. This leads to another highlight of this study is that hepatectomy is as first-line therapy for patients with HCC and PVTT, which makes the conclusion more believable. The study also indicates that even patients with HCC and PVTT who do not respond to any neoadjuvant therapy might benefit from surgical resection. However, 2 things might be considered to draw a fine conclusion according to this study.

Firstly, it is believed that adjuvant therapy (such as sorafenib) is needed for patients with HCC and PVTT after surgery, but the authors did not mention any adjuvant therapy in the study. If patients received surgery also received adjuvant therapy, then the conclusion might be resection plus adjuvant therapy is more favorable for patients with HCC and PVTT. Furthermore, surgical procedure could be different including hepatectomy with resection of the branch of the portal vein, en bloc resection with reconstruction of the portal vein or thrombectomy.^6^ The statistics of surgical procedure might enlighten surgeon to choose the appropriate procedure.

Secondly, the author concluded that BCLC C patients without extrahepatic spread but with intrahepatic portal invasion, liver resection, if feasible, was followed by better Overall survival and PFS compared with sorafenib. However, we could see that many differences were observed in the baseline characteristics of the 2 groups. Especially, the tumor burden of the 2 groups are significantly different, as “bilobar disease was found in 11.9% of SURG cases and 38.3% of SOR subjects (*P* < 0.001)... The median number of nodules was one (IQR 1–1) and two (1–4) for SURG and SOR, respectively (*P* =0.004),” which would seriously affect the reliability of the conclusion. As a result, it might be more exact to conclude surgical resection is prior to sorafenib for BCLC C patients without extrahepatic spread but with only 1 tumor node, intrahepatic portal invasion and Child-Pugh A grade although further statistic would be needed.

Moreover, it is believed that selected patients with HCC and PVTT (like Portal vein thrombosis 1 and 2) would benefit from surgical resection, but surgery for patients with HCC and PVTT 3 (first-order branches of the portal vein) is controversial. So, the survival pattern of patients with HCC and PVTT 3 who received resection might be discussed as it could shed more light on this issue. According to the analysis above, 4 subgroups could be added to elucidate these questions—group A: patients with HCC and PVTT 1 who received surgery (n = 66); group B: patients with HCC and PVTT 3 who received surgery (n = 81); group C: patients with HCC and PVTT 1 who received sorafenib (n = 14); and group D: patients with HCC and PVTT 3 who received sorafenib (n = 82). Then, propensity score matching based on the baseline characters (including tumor burden, Child-Pugh grade, etc.) could be done between groups A and C, groups B and D. Finally, the Overall survival and PFS could be compared between groups A and C, groups B and D so that a fair conclusion could be made to further guide the surgical intervention for BCLC C patients without extrahepatic spread but with intrahepatic portal invasion.

## References

[1] Tremosini S Reig M de Lope CR. Treatment of early hepatocellular carcinoma: towards personalized therapy. Dig Liver Dis. 2010;42(suppl 3):S242–S248. 20547310 10.1016/S1590-8658(10)60512-9

[2] Reig M Forner A Rimola J. BCLC strategy for prognosis prediction and treatment recommendation: the 2022 update. J Hepatol. 2022;76:681–693. 34801630 10.1016/j.jhep.2021.11.018 PMC8866082

[3] Cheng AL Qin S Ikeda M. Updated efficacy and safety data from IMbrave150: atezolizumab plus bevacizumab vs. sorafenib for unresectable hepatocellular carcinoma. J Hepatol. 2022;76:862–873. 34902530 10.1016/j.jhep.2021.11.030

[4] Famularo S Donadon M Cipriani F. Hepatectomy versus sorafenib in advanced nonmetastatic hepatocellular carcinoma: a real-life multicentric weighted comparison. Ann Surg. 2022;275:743–752. 35081572 10.1097/SLA.0000000000005373

[5] Liang L Chen TH Li C. A systematic review comparing outcomes of surgical resection and non-surgical treatments for patients with hepatocellular carcinoma and portal vein tumor thrombus. HPB (Oxford). 2018;20:1119–1129. 30056066 10.1016/j.hpb.2018.06.1804

[6] Sakamoto K Nagano H. Surgical treatment for advanced hepatocellular carcinoma with portal vein tumor thrombus. Hepatol Res. 2017;47:957–962. 28618075 10.1111/hepr.12923

